
JOURNAL INFORMATION
==============================
NLM Title Abbreviation: ScientificWorldJournal
Journal ID: ScientificWorldJournal
NLM Journal ID: 101131163
Journal ID: TSWJ

The Scientific World Journal

ISSN: 2356-6140
EISSN: 1537-744X
Publisher: Wiley

ARTICLE INFORMATION
==============================
PMCID: PMC6009419
PMID: 29973837
DOI: 10.1100/tsw.2002.115
Article ID: 705438
Article version: 1
Subjects: Editorial

Announcing Topics in Bone Biology — A Three-Volume Reference Work to be Published by TheScientificWorld

Allen Anne Vindenes *
TheScientificWorld,
*Anne Vindenes Allen: aallen@thescientificworld.com
Publication date: 2002
Electronic publication date: 2002 Jan 16
Volume: 2
First page: 131
Last page: 133
Received 2002 Jan 2; Accepted 2002 Jan 2
Copyright: Copyright © 2002 Anne Vindenes Allen.
Copyright year: 2002
License: This is an open access article distributed under the Creative Commons Attribution License, which permits unrestricted use, distribution, and reproduction in any medium, provided the original work is properly cited.
License URL: https://creativecommons.org/licenses/by/3.0/

Keywords: osteoblast orientation and differentiation, osteoblast activity, bone formation, biomechanical aspects, bone resorption, functional skeletal tissues

==============================
Publisher's Note
Topics in Bone Biology: Bone Formation
*TheScientificWorldJOURNAL* (2002) 2, 131–133
ISSN 1537-744X; DOI 10.1100/tsw.2002.115

### Announcing *Topics in Bone Biology* – A Three-Volume Reference Work to be Published by TheScientificWorld

Anne Vindenes Allen
*TheScientificWorld*

*Received January 2, 2002; Accepted January 2, 2002; Published January 16, 2002*

**KEY WORDS:** osteoblast orientation and differentiation, osteoblast activity, bone formation, biomechanical aspects, bone resorption, functional skeletal tissues

**DOMAINS:** bone biology, cell biology, endocrinology, nephrology, metabolic diseases

TheScientificWorld is pleased to announce the forthcoming publication of *Topics in Bone Biology*, a major new reference work in three volumes. The Series Editors are Professors Felix Bronner, Ph.D. and Mary C. Farach-Carson, Ph.D. Professor Bronner is Professor Emeritus at the University of Connecticut Health Center. He is Principal Editor for the Bone Biology domain of *TheScientificWorldJOURNAL* and is a world authority on bone biology with numerous publications and books to his credit. Professor Farach-Carson is Professor at the Department of Biological Science at the University of Delaware and is Editorial Board Member for the Bone Biology domain of *TheScientificWorldJOURNAL* and for numerous other peer-reviewed publications. Her work is within the fields of bone and extracellular matrix biology, calcitropic hormones, and calcium channels, focusing on the role of calcitropic hormones in regulating the bone remodeling process.

**Volume 1. Bone Formation**

Volume 1 of *Topics in Bone Biology* deals with the aspects of Bone Formation. It is edited by Professors Bronner and Farach-Carson; in Volumes 2 and 3 they will be joined by co-editors with expertise in the volume topics.

Bone formation is a complicated multiple step that involves genomic, cellular, endocrine, paracrine, and autocrine events that in turn are modulated by nutritional and cultural factors. The current volume, authored by basic and clinical scientists, summarizes new knowledge of the many steps that lead to the formation of bone. By incorporating established knowledge concerning these processes, the chapters provide a basis for further research and ultimate therapy.

The Volume, authored by internationally known authorities, should be of interest to students, researchers, and resident physicians, as well as medical specialists – internists, endocrinologists, nephrologists, orthopaedists, to name just a few – and to dental, paramedical, and veterinary practicioners. It should prove of particular value to those entering the field of bone biology and those who wish to become familiar with up-to-date information in a particular area of bone research.

Email: aallen@thescientificworld.com
©2002 with author.

Topics and authors for Volume 1 are:

- Osteoblast Origin and Differentiation: J.E. Aubin and M.D. McKee
  University of Toronto and McGill University

- Mineralized Matrix Formation by Osteoblasts: J.E. Puzas
  University of Rochester

- Genetics and Mutations Affecting Osteoblast Formation: A.C. Karaplis
  Lady Davis Institute for Medical Research, Montreal

- Diseases of Inadequate Bone Formation: T.J. Martin and E. Seeman
  St. Vincent's Institute of Medical Research, Fitzroy and University of Melbourne

- *In Vitro* Regulation of Osteoblast Activity: M.C. Farach-Carson and N.J. Karin
  University of Delaware

- Endocrine, Autocrine, and Paracrine Regulation of Bone Formation: M. Hurley and Joseph Lorenzo
  University of Connecticut

- Pharmaceuticals Targeting the Osteoblast: M. Peterlik
  University of Vienna

- Diseases of Excess Bone Formation: B.R. Olsen and W. McLean
  Harvard University

- Biomechanical Aspects of Bone Formation: C.H. Turner
  Indiana University

Further, planned volumes include:

**Volume 2. Bone Resorption**

Bone resorption is a process that ensures removal of previously formed bone with new bone and therefore is essential to bone growth, as well as to the maintenance of a stable bone mass balance. When bone resorption exceeds formation, there is loss of bone mass, as encountered in osteoporosis and osteopenia. Excess bone resorption is characteristic of certain diseases, e.g., lytic bone cancer or ectopic bone formation. This Volume, authored by basic and clinical scientists, provides an up-to-date synopsis of recent progress in understanding normal and pathological bone resorption, along with established knowledge that is the basis for further progress and eventual therapy.

**Volume 3. Engineering of Functional Skeletal Tissues**

Congenital defects, injury, and disease are conditions that may be significantly helped by skeletal and dental prostheses. Much research is still needed before prostheses adequately simulate natural function, even though dental implants and knee and hip replacements are already effective. Yet the basis for long-term function or dysfunction of theses prostheses needs much further understanding, as does the development of other prostheses. The current Volume, authored by bioengineers and basic and clinical scientists, addresses questions of design and biocompatibility. In addition, future tissue replacement depends on understanding of how cells function in organs and how cellular function is integrated into organ function. It is this type of integrated knowledge that the current volume will provide.

All Volumes will be published electronically (*Volume 1. Bone Formation* has assigned ISBN 1-931831-02-5), and chapters will also be accessible through the Bone Biology domain of *TheScientificWorldJOURNAL*. TheScientificWorld is currently investigating the possibility of providing this authoritative work as a printed book, and further information will be posted on *TheScientificWorldJOURNAL* web site at www.thescientificworld.com as it becomes available. For pricing, ordering, and other information about *Topics in Bone Biology*, contact the Publisher at MemberService@thescientificworld.com

**Anne Vindenes Allen**
Editorial Director
TheScientificWorld U.K.

**This article should be referenced as follows:**

Allen, A.V. (2002) Announcing *Topics in Bone Biology* – a three-volume reference work to be published by TheScientificWorld. In Topics in Bone Biology: Bone Formation. *TheScientificWorldJOURNAL* **2**, 131–133.
